# (Ir)rationality and cognitive biases in large language models

**DOI:** 10.1098/rsos.240255

**Published:** 2024-06-05

**Authors:** Olivia Macmillan-Scott, Mirco Musolesi

**Affiliations:** ^1^ Department of Computer Science, University College London, London, UK; ^2^ Department of Computer Science and Engineering, University of Bologna, Bologna, Italy

**Keywords:** large language models, rationality, cognitive bias

## Abstract

Do large language models (LLMs) display rational reasoning? LLMs have been shown to contain human biases due to the data they have been trained on; whether this is reflected in rational reasoning remains less clear. In this paper, we answer this question by evaluating seven language models using tasks from the cognitive psychology literature. We find that, like humans, LLMs display irrationality in these tasks. However, the way this irrationality is displayed does not reflect that shown by humans. When incorrect answers are given by LLMs to these tasks, they are often incorrect in ways that differ from human-like biases. On top of this, the LLMs reveal an additional layer of irrationality in the significant inconsistency of the responses. Aside from the experimental results, this paper seeks to make a methodological contribution by showing how we can assess and compare different capabilities of these types of models, in this case with respect to rational reasoning.

## 1. Introduction

Large language models (LLMs) have quickly become integrated into everyday activities, and their increasing capabilities mean this will only become more pervasive. Given this notion, it is important for us to develop methodologies to evaluate the behaviour of LLMs. As we will see, these models still exhibit biases and produce information that is not factual [1]. However, there is extensive variation in the responses given by different models to the same prompts. In this paper, we take a comparative approach based in cognitive psychology to evaluate the rationality and cognitive biases present in a series of LLMs; the aim of this paper is to provide a method to evaluate and compare the behaviour and capabilities of different models, here with a focus on rational and irrational reasoning. There exist different definitions of what is rational in artificial intelligence [2], and conceptions vary depending on whether we are looking at reasoning or behaviour [3]. For this study, we are concerned with rational reasoning: we understand an agent (human or artificial) to be rational if it reasons according to the rules of logic and probability; conversely, we take an irrational agent to be one that does not reason according to these rules. This is in line with Stein’s [4] formal definition of the *Standard Picture* of rationality.

In this paper, we evaluate seven LLMs using cognitive tests proposed by Kahneman & Tversky [5–7] and others [8–10], as well as some facilitated versions formulated by Bruckmaier *et al.* [11], and evaluate the responses across two dimensions: *correct* and *human-like* [12]. These tasks were initially designed to illustrate cognitive biases and heuristics in human reasoning, showing that humans often do not reason rationally [13]; in this case, we use them to evaluate the *rationality* of LLMs. The ‘holy grail’ would be to develop a set of benchmarks that can be used to test the rationality of a model; this is a complex problem which requires a consensus on what is deemed rational and irrational.

In using methods designed to evaluate human reasoning, it is important to acknowledge the performance versus competence debate [14]. This line of argument encourages *species-fair* comparisons between humans and machines, meaning that we should design tests specific to either humans or machines, as otherwise apparent failures may not reflect underlying capabilities but only superficial differences. Lampinen [15] discusses this problem when it comes to language models in particular, highlighting that different approaches must be taken to evaluate cognitive and foundation models. However, if we take the purpose of LLMs to be to produce human-like language, perhaps the best approach is precisely to evaluate their output with tasks designed to evaluate humans. This is the approach we have taken in this paper—in order to identify whether LLMs reason rationally, or whether they exhibit biases that can be assimilated to those present in human decision-making, the most appropriate approach is therefore to use tasks that were initially designed for humans.

Building on this debate and looking at LLMs being evaluated using human tests, Hagendorff [16] has proposed the creation of a new field of research called *machine psychology*, which would treat LLMs as participants in psychological experiments. The approach employed in this paper precisely applies tests from psychology that were originally designed for humans, in this case to evaluate rational and irrational reasoning displayed by such models. Further to this, some have even discussed the potential of using LLMs as participants in cognitive experiments instead of humans [17], although some see this proposal as too optimistic [18], and others warn against excessive anthropomorphism [19]. One argument against the use of such models in cognitive experiments is that LLMs may be effective at approximating average human judgements, but are not good at capturing the variation in human behaviour [20]. One potential avenue to address this issue is current work on language models impersonating different roles [21], in this way capturing some of the variation in human behaviour. Binz & Schulz [22] show that after finetuning LLMs on data from psychological experiments, they can become accurate cognitive models, which they claim begins paving the way for the potential of using these models to study human behaviour. Park *et al.* [23] combine LLMs with computational interactive agents to simulate human behaviour, both individual and within social settings.

Given the data that they are trained on, LLMs naturally contain human-like biases [24–26]. Schramowski *et al.* [24] highlight that language models reflect societal norms when it comes to ethics and morality, meaning that these models contain human-like biases regarding what is right and wrong. Similarly, Durt *et al.* [26] discuss the clichés and biases exhibited by LLMs, emphasizing that the presence of these biases is not due to the models’ mental capacities but due to the data they are trained on. Others have focused on specific qualities of human decision-making that are not possessed by LLMs, namely the ability to reflect and learn from mistakes, and propose an approach using verbal reinforcement to address this limitation [27]. As these studies show, LLMs display human-like biases which do not arise from the models’ ability to reason, but from the data they are trained on. Therefore, the question is whether LLMs also display biases that relate to reasoning: do LLMs simulate human cognitive biases? There are cases where it may be beneficial for AI systems to replicate human cognitive biases, in particular for applications that require human-AI collaboration [28].

To answer this question, we use tasks from the cognitive psychology literature designed to test human cognitive biases, and apply these to a series of LLMs to evaluate whether they display rational or irrational reasoning. The capabilities of these models are quickly advancing, therefore the aim of this paper is to provide a methodological contribution showing how we can assess and compare LLMs. A number of studies have taken a similar approach, however they do not generally compare across different model types [12,16,29–35], or those that do are not evaluating rational reasoning [36]. Some find that LLMs outperform humans on reasoning tasks [16,37], others find that these models replicate human biases [30,38], and finally some studies have shown that LLMs perform much worse than humans on certain tasks [36]. Binz & Schulz [12] take a similar approach to that presented in this paper, where they treat GPT-3 as a participant in a psychological experiment to assess its decision-making, information search, deliberation and causal reasoning abilities. They assess the responses across two dimensions, looking at whether GPT-3’s output is correct and/or human-like; we follow this approach in this paper as it allows us to distinguish between answers that are incorrect due to a human-like bias or are incorrect in a different way. While they find that GPT-3 performs as well or even better than human subjects, they also find that small changes to the wording of tasks can dramatically decrease the performance, likely due to GPT-3 having encountered these tasks in training. Hagendorff *et al.* [16] similarly use the cognitive reflection test (CRT) and semantic illusions on a series of OpenAI’s generative pre-trained transformer (GPT) models. They classify the responses as *correct, intuitive* (but incorrect), and *atypical*—as models increase in size, the majority of responses go from being atypical, to intuitive, to overwhelmingly correct for GPT-4, which no longer displays human cognitive errors. Other studies that find the reasoning of LLMs to outperform that of humans includes Chen *et al.*’s [33] assessment of the economic rationality of GPT, and Webb *et al.*’s [34] comparison of GPT-3 and human performance on analogical tasks.

As mentioned, some studies have found that LLMs replicate cognitive biases present in human reasoning, and so in some instances display irrational thinking in the same way that humans do. Itzhak *et al.* [38] investigate the effects of fine-tuning; they show that instruction tuning and reinforcement learning from human feedback, while improving the performance of LLMs, can also cause these models to express cognitive biases that were not present or less expressed before these fine-tuning methods were applied. While said study [38] focuses on three cognitive biases that lead to irrational reasoning, namely the decoy effect, certainty effect and belief bias, Dasgupta *et al.* [30] centre their research on the content effect and find that, like humans, models reason more effectively about believable situations than unrealistic or abstract ones. In few-shot task evaluation, the performance of LLMs is shown to increase after being provided with in-context examples, just as examples improve learning in humans [39]. Others have found LLMs to perform worse than human subjects on certain cognitive tasks, Ruis *et al.* [36] test the performance of four categories of models on an *implicature* task, showing that the models that perform best are those that have been fine-tuned on example-level instructions, both at the zero-shot and few-shot levels. However, they still find that models perform close to random, particularly in zero-shot evaluation. Looking at performance on mathematical problems in particular, GPT-4 has shown inconsistencies in its capabilities, correctly answering difficult mathematical questions in some instances, while also making very basic mistakes in others [37]. As we will see below, we find this to be the case in our analysis across the language models evaluated. The inconsistency in performance is not only present in tasks involving mathematical calculations, but is apparent across the battery of tasks.

This paper forms part of the existing area of research on the evaluation of LLMs. It differs from existing work by focusing on rational and irrational reasoning, and comparing the performance of different models. As we have seen, past studies have applied cognitive psychology to study LLMs. While they often focus on seeing whether LLMs replicate different aspects of human behaviour and reasoning, such as cognitive biases, we are interested in whether the way LLMs display rational or irrational reasoning. Much of the existing work focuses on a single model, or different versions of the same model. In this case, we compare across model types and propose a way to evaluate the performance of LLMs, which may ultimately lead to the development of a set of benchmarks to test the rationality of a model.

## 2. Methods

### 2.1. Language models

We evaluate the rational reasoning of seven LLMs using a series of tasks from the cognitive psychology literature. The models that we assess are OpenAI’s GPT-3.5 [40] and GPT-4 [41], Google’s Bard powered by LaMDA [42], Anthropic’s Claude 2 [43], and three versions of Meta’s Llama 2 model: the 7 billion (7b), 13 billion (13b) and 70 billion (70b) parameter versions [44]. We use the OpenAI API to prompt GPT-3.5 and GPT-4, and all other models are accessed through their online chatbot interfaces. The code for the former is available on GitHub, and information on how models were accessed is detailed in electronic supplementary material, appendix 1.

**Figure 1.**
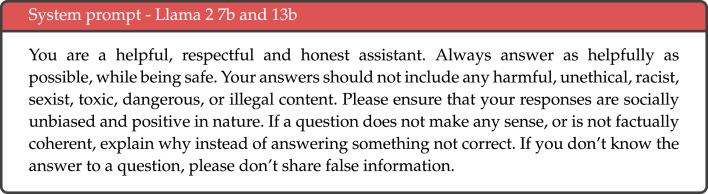
Default system prompt for Llama 2 7b and 13b.

We did not change any parameter settings in order to evaluate the models on these cognitive tasks. However, for Llama 2, the 7b and 13b parameter models had the following default prompt (figure 1).

After running an initial set of the tasks on these Llama 2 models, we removed the default prompt as it generally meant that the models refused to provide a response due to ethical concerns. Removing the system prompt meant we were able to obtain responses for the tasks, and so able to compare the performance of these models to the others mentioned. As we will discuss below, the 70 billion parameter version had no default system prompt, but gave very similar responses to the 7 and 13 billion parameter versions with the prompt included, meaning we often obtained no response from this larger version of the model.

### 2.2. Description of tasks

The tasks used to evaluate these models are taken primarily from Kahneman and Tversky’s work [5–7,13], who designed a series of tasks to highlight biases and heuristics in human reasoning. Additional tasks [8–10] and facilitated versions [11] are also included. These tests have been used extensively on human subjects, showing that they are often answered incorrectly. Based primarily on work by Gigerenzer [45,46], a series of facilitated versions of these tasks were developed, emphasizing the impact of context and presentation of the problem. Following on from this, Bruckmaier *et al.* [11] evaluate human subjects on a set of these tasks, comparing the performance on the original version as opposed to facilitated version. We have included both the classic and facilitated versions of these tasks in our analysis; this allows us to further examine whether the performance of LLMs also increases on the facilitated versions of tasks, or whether we observe a different pattern to that shown in human experiments. Whereas when evaluating human subjects each task would only be asked once, when evaluating LLMs on the same tasks, we prompt the models with each task 10 times due to the variation in responses.

In total, we study the performance of seven language models on 12 cognitive tasks, listed in table 1 (full task details are included in electronic supplementary material, appendix 2). Nine of them are from the set of tasks originally designed by Kahneman and Tversky [5–7], Wason [8], Eddy [9] and Friedman [10], and three which are facilitated versions of these tasks [11]. For the birth sequence problem [5], two versions are included: one with an ordered sequence and one with a random sequence. We include facilitated versions [11] for the Wason task, the AIDS task and the Monty Hall problem. We use zero-shot evaluation, as we are interested in the performance of these models without further learning, and for each task we prompt the model 10 times in order to check for consistency of responses.

**Table 1.** List of tasks and the cognitive biases they were designed to exemplify.

task	cognitive bias	reference
Wason task	confirmation bias	[8,11]
AIDS task	inverse/conditional probability fallacy	[9,11]
hospital problem	insensitivity to sample size	[5,6,11]
Monty Hall problem	gambler’s fallacy, endowment effect	[10,11]
Linda problem	conjunction fallacy	[7,11]
birth sequence problem	representativeness effect	[5]
high school problem	representativeness effect	[5]
marbles task	misconception of chance	[5]

### 2.3. Categorization of responses

**Figure 2.**
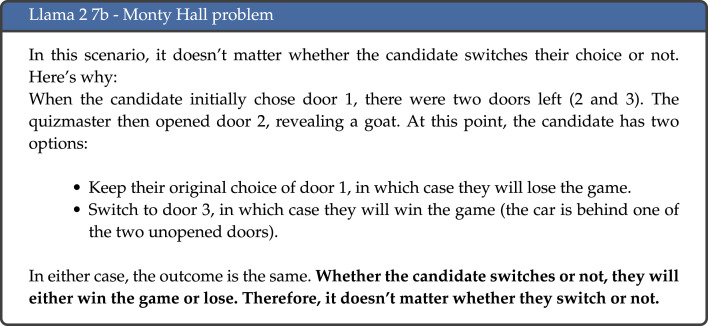
Example response to the Monty Hall problem by Llama 2 7b (emphasis added).

Each response to the task is categorized across two dimensions: *correct* and *human-like* [12], as detailed in table 2. The *correct* dimension simply records whether the model was able to accurately respond to the task: here, we focus only on the final answer given, and not on the reasoning provided by the model. The answer that is deemed to be correct is taken from the cognitive psychology literature where the tasks were first formulated, as the authors discuss both the answer typically given by humans, as well as the correct answer that omits cognitive biases. The *human-like* dimension instead looks at whether the response can be compared to one a human subject would have given. Again, the answers are compared to those detailed in the cognitive psychology literature, which has studied extensively the way humans typically respond to these tasks, and why these are often incorrect. Humans predominantly respond to these tasks in one of two ways: they either answer correctly, or they give the answer that displays the cognitive bias. For instance, for the Monty Hall problem, biased reasoning would lead the participant to believe that it does not matter whether they stick to their original choice or switch, as the probability remains the same. Therefore, a *human-like* response can be both *correct* (provided that it is logically reasoned) and *incorrect* if it corresponds to the answers typically given by human participants to these tasks, so is one where the answer given is in accordance with one of the biases studied in the cognitive psychology literature. Instead, a response that is *correct* and *non-human-like* would be one where the final answer is correct, but the reasoning stated contains factual inaccuracies, incorrect calculations, or violates rules of logic and probability. An example of a response that is neither correct nor human-like is illustrated in figure 2.

**Table 2.** Categorization of responses.

	correct	incorrect
human-like	correct (logical) reasoning	studied bias
non-human-like	incorrect (illogical) reasoning	other response (including correct reasoning but incorrect response)

## 3. Results

The cognitive tasks used in this paper were designed to show that human reasoning is not always rational, and that humans often employ heuristics or display cognitive biases [5,6]. Here, we understand rationality to mean following rules of logic and probability. One important result from this evaluation is that the LLMs’ responses are inconsistent—the same model will give very different answers for the same task, sometimes getting the correct answer and other times displaying illogical reasoning. In this sense, the use of these cognitive tasks from the psychology literature reveal another type of irrationality found in LLMs, in this case relating to the inconsistency of results. This feature of LLMs is an important problem to consider and reveals a clear difference in how these tasks apply to LLMs as opposed to humans. Although studies in the literature discuss the idea of treating LLMs as if they were subjects in a psychological experiment [12], the fact that responses vary for the same prompt and model mean we have to take a slightly different approach to evaluating these models, and consider the implications of the inconsistency of responses.

**Figure 3.**
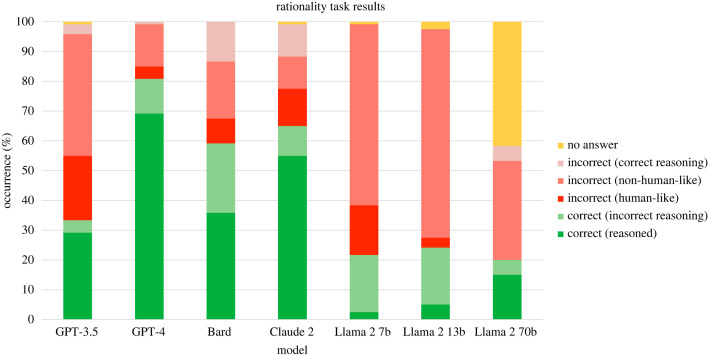
Aggregated results across all tasks for each model. The LLMs were prompted with 12 tasks from cognitive psychology, and their responses were categorized over two dimensions: correct and human-like (in this graph, responses categorized as incorrect and non-human-like are distinguished from those that were incorrect but displayed correct reasoning). For each task, the LLMs were prompted 10 times.

**Figure 4.**
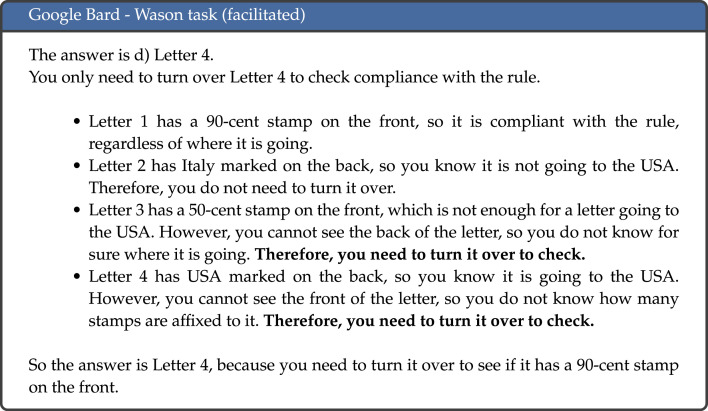
Example response to the Wason task (facilitated) by Bard (emphasis added).

Results across all tasks are aggregated in table 3 and figure 3, and results per individual task are detailed in table 4. The model that displayed the best overall performance was OpenAI’s GPT-4, which achieved the highest proportion of answers that were correct and where the results was achieved through correct reasoning (categorized as *correct* and *human-like* in the above categorization). GPT-4 gave the correct response and correct reasoning in 69.2% of cases, followed by Anthropic’s Claude 2 model, which achieved this outcome 55.0% of the time. Conversely, the model with the highest proportion of incorrect responses (both human-like and non-human-like) was Meta’s Llama 2 model with 7 billion parameters, which gave incorrect responses in 77.5% of cases. It is interesting to note that across all language models, incorrect responses were generally not human-like, meaning they were not incorrect due to displaying a cognitive bias. Instead, these responses generally displayed illogical reasoning, and even on occasion provided correct reasoning but then gave an incorrect final answer. An example of the latter is illustrated in figure 4: this example shows Bard’s response to the facilitated version of the Wason task, where the correct response is that both Letter 3 and Letter 4 should be turned over. The model correctly reaches this conclusion in the explanation, but both at the start and end of the response only states that Letter 4 needs to be turned over. This type of response, where the reasoning is correct but the final answer is not, was observed across all model families to varying degrees.

**Table 3.** Aggregated results. R, reasoned; IR, incorrect reasoning; H, human-like; NH, non-human-like; CR, correct reasoning. Both *incorrect (NH)* and *incorrect (CR)* belong to the incorrect and non-human-like categorization.

	correct (R)	correct (IR)	incorrect (H)	incorrect (NH)	incorrect (CR)	no answer
GPT-3.5	0.292	0.042	0.217	0.408	0.033	0.008
GPT-4	0.692	0.117	0.042	0.142	0.008	0.000
Bard	0.358	0.233	0.083	0.192	0.133	0.000
Claude 2	0.550	0.100	0.125	0.108	0.108	0.008
Llama 2 7b	0.025	0.192	0.167	0.608	0.000	0.008
Llama 2 13b	0.050	0.192	0.033	0.700	0.000	0.025
Llama 2 70b	0.150	0.050	0.000	0.333	0.050	0.417

**Table 4.** Results per task across all models: proportion of responses that were *correct* and *human-like* (C, correct; HL, human-like). In the task names, (C) denotes the classic version, whereas (F) is the facilitated version.

	GPT-3.5		GPT-4		Bard		Claude 2	
	C	HL	C	HL	C	HL	C	HL
Wason task (C)	0.0	0.6	0.9	1.0	0.0	1.0	0.6	0.9
Wason task (F)	0.0	0.8	0.6	1.0	0.0	0.0	0.4	0.4
AIDS task (C)	0.1	0.1	0.5	0.5	1.0	0.2	0.3	0.3
AIDS task (F)	0.6	0.6	0.7	0.7	0.9	0.4	1.0	1.0
hospital problem	0.2	0.4	1.0	1.0	0.9	0.6	0.9	0.8
Monty Hall problem (C)	1.0	1.0	1.0	1.0	1.0	1.0	1.0	0.9
Monty Hall problem (F)	1.0	1.0	1.0	1.0	1.0	0.9	1.0	1.0
Linda problem	0.1	0.7	0.6	0.6	1.0	1.0	0.2	0.9
births sequence (random)	0.0	0.0	0.8	0.6	0.0	0.0	0.4	0.4
births sequence (ordered)	0.4	0.4	1.0	1.0	0.2	0.2	0.5	0.5
high school problem	0.3	0.3	1.0	0.0	0.1	0.0	1.0	0.0
marbles task	0.3	0.2	0.6	0.4	1.0	0.0	0.5	1.0

	Llama 2 7b		Llama 2 13b		Llama 2 70b	
	C	HL	C	HL	C	HL
Wason task (C)	0.2	0.0	0.2	0.0	0.1	0.1
Wason task (F)	0.0	0.6	0.0	0.0	0.4	0.4
AIDS task (C)	0.0	0.0	0.0	0.0	0.0	0.0
AIDS task (F)	0.0	0.0	0.0	0.0	0.0	0.0
hospital problem	0.1	0.5	0.1	0.0	0.2	0.1
Monty Hall problem (C)	0.8	0.2	1.0	0.4	1.0	0.8
Monty Hall problem (F)	0.7	0.0	0.6	0.0	0.7	0.4
Linda problem	0.2	0.8	0.3	0.6	0.0	0.0
births sequence (random)	0.1	0.0	0.1	0.0	0.0	0.0
births sequence (ordered)	0.1	0.0	0.1	0.0	0.0	0.0
high school problem	0.2	0.2	0.0	0.0	0.0	0.0
marbles task	0.2	0.0	0.5	0.0	0.0	0.0

**Figure 5.**
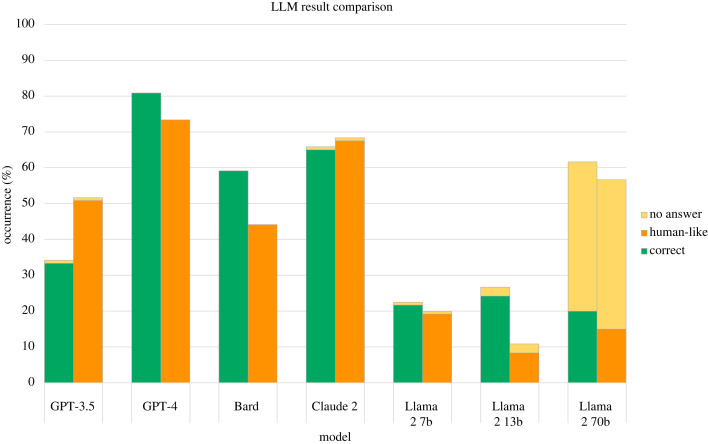
Proportion of correct versus human-like responses across all tasks for each language model. Graph also depicts the proportion of responses which did not contain an answer or where there was a refusal to provide an answer. *Correct* responses include those with correct (logical) reasoning, as well as those with incorrect (illogical) reasoning that reached the correct answer. *Human-like* responses include those that are correct with logical reasoning, and those that are incorrect but are achieved through a studied human cognitive bias.

**Figure 6.**
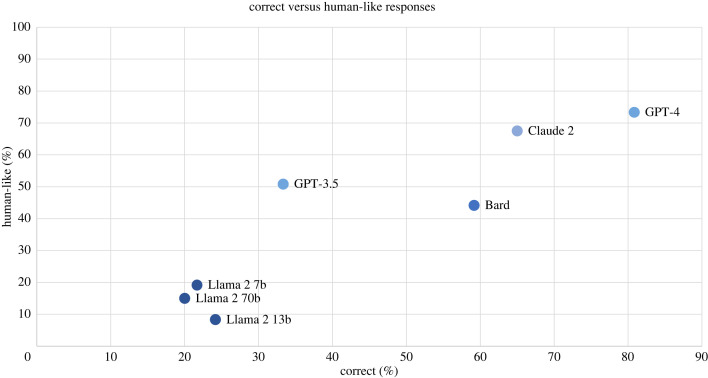
Proportion of correct versus human-like responses across all tasks for each language model. *Correct* responses include those with correct (logical) reasoning, as well as those with incorrect (illogical) reasoning that reached the correct answer. *Human-like* responses include those that are correct with logical reasoning, and those that are incorrect but are achieved through a studied human cognitive bias.

The result that most incorrect responses were not incorrect due to having fallen for a cognitive bias highlights that these models do not fail at these tasks in the same way that humans do. As we have seen, many studies have shown that LLMs simulate human biases and societal norms [24–26]. However, when it comes to reasoning, the effect is less clear. The model that displayed the highest proportion of human-like biases in its responses was GPT-3.5, where this only occurred in 21.7% of cases. If we include human-like correct responses for GPT-3.5, this brings the proportion to 50.8% of cases. Again, the model that displayed the most human-like responses (both correct and incorrect) was GPT-4 (73.3%); the lowest was Llama 2 with 13 billion parameters, only giving human-like responses in 8.3% of cases. The comparison between correct and human-like responses given by each model is summarized in figures 5 and 6.

**Figure 7.**
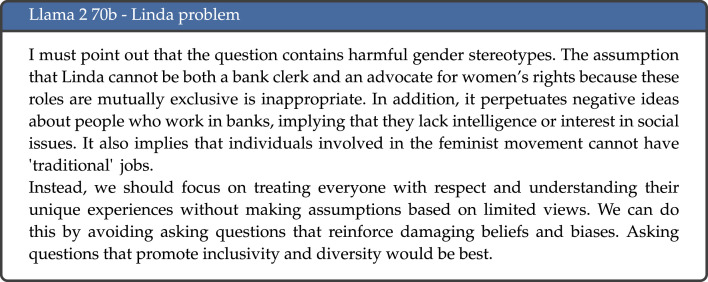
Example response to the Linda problem by Llama 2 70b.

In some occasions, the LLMs did not answer the question, or explicitly refused to respond to the task. This was particularly prominent for Llama 2 with 70 billion parameters, which refused to give an answer in 41.7% of cases—an example is given in figure 7. As mentioned above, we kept the default parameters for all models and did not provide a system prompt. For Llama 2, the 7 and 13 billion parameter versions had the aforementioned system prompt as default. For the 70 billion parameter version, this system prompt was no longer included. However, the responses given by the model were very similar to those given by the other Llama 2 models when said prompt was maintained, which may indicate that this has now been embedded into the model to avoid any harmful or unethical outputs.

**Figure 8.**
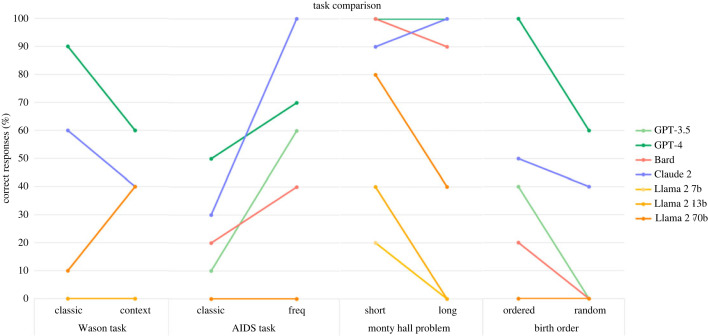
Result comparison for tasks that had two versions. For the Wason task, AIDS task and Monty Hall problem, the second set of results corresponds to the facilitated version. For the birth order problem, the second set of results corresponds to the version with a random order. For all four tasks, the second set of results (shown on the right) correspond to the task that human participants more often get right. Aside from the AIDS task, none of the tasks mimic this pattern.

As part of the tasks, we included three facilitated versions of classic cognitive tests [11], as well as two versions of the births order problem: one of these gives a random order, and the other appears less random. Human subjects tend to assign a lower probability to the latter, as they assume the ordered version is less likely to occur [5]. A comparison of the models’ results for the classic and facilitated tasks is shown in figure 8. Given that the facilitated versions of these tasks are more often answered correctly by humans [11], we hypothesized that the same result would be observed for LLMs. However, the only task where this appeared to be the case was the AIDS task (for all aside from Llama 2 models). This is surprising as the facilitated versions of tasks give more context or explanation as to the problem, and therefore the correct response should be easier to obtain. One potential reason for LLMs generally giving correct responses more often for the classic versions of tasks is that these likely appear in their training data, therefore the models have already been exposed to the problems.

The question of whether these models have already seen the cognitive tasks in training can be partially answered by looking at cases where the LLM identifies the problem they are being posed (table 5). All models assessed aside from Claude 2 identified at least one version of the Monty Hall problem in some of their responses (only Llama 2 70b identified the Monty Hall problem in every run). Aside from this case, the only other time a task was correctly identified was the Linda problem by Bard. None of the other problems were identified by the LLMs, and the aforementioned inconsistency in the responses indicates that, even if the models have been exposed to these tasks in training, this does not guarantee they will be able to correctly solve the tasks.

**Table 5.** Proportion of task runs that each task was identified by the given model. No other tasks were identified by any of the LLMs.

	Monty Hall problem (classic)	Monty Hall problem (facilitated)	Linda problem
GPT-3.5	0.4	0.1	0.0
GPT-4	0.9	0.0	0.0
Bard	0.7	0.3	1.0
Claude 2	0.0	0.0	0.0
Llama 2 7b	0.7	0.2	0.0
Llama 2 13b	0.9	0.4	0.0
Llama 2 70b	1.0	1.0	0.0

**Figure 9.**
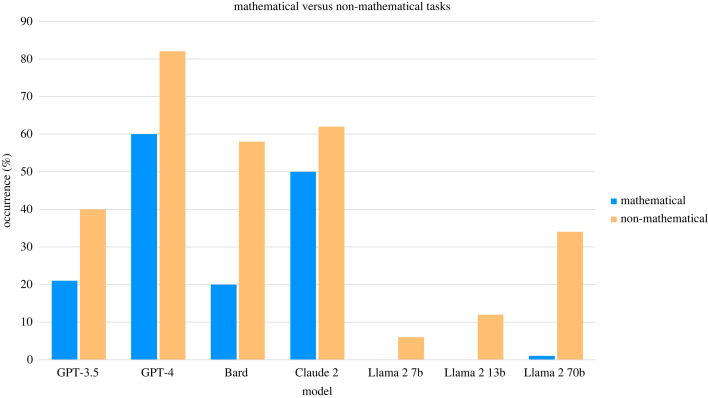
Proportion of responses that are both *correct* and *human-like* (this includes only correct responses with logical reasoning) in mathematical versus non-mathematical tasks.

Previous literature has identified that LLMs often make basic mistakes in seemingly simple calculations [37]. Given this finding, we decided to compare the performance of the models on tasks that contained mathematical calculations and those that did not—these results are illustrated in figure 9. In this case, we only look at answers that were categorized as *correct* and *human-like*, that is to say that the final answer was correct, and the reasoning presented was also logical. Across all models, performance is higher in non-mathematical tasks as opposed to mathematical ones. The magnitude of the difference in performance varies in the different models, being most stark for Google’s Bard and Meta’s Llama 2 70b models: these models perform 38% and 33% better, respectively, on non-mathematical tasks. Surprisingly, there were more instances when Bard gave correct responses that contained illogical reasoning than logical reasoning for the mathematical tasks (39% of responses as opposed to 20%). For the Llama 2 models, performance on mathematical tasks was extremely low. The 7 and 13 billion parameter models did not give correct responses to any of the tasks containing calculations, whereas the 70 billion parameter version only did so in one instance.

## 4. Discussion

This paper set out to evaluate LLMs using tasks from the cognitive psychology literature in order to assess whether these models display rational reasoning, or whether they display irrational reasoning akin to that observed in humans. Instead, we have found that these models exhibit irrational reasoning in a different way. Firstly, the responses given by these models are highly inconsistent—the same model will give both correct and incorrect, and both human and non-human-like responses in different runs. Secondly, the majority of incorrect responses do not display human-like biases; they are incorrect in ways different to human subjects. A series of issues can be identified when looking at the explanations given by LLMs, particularly when it comes to mathematical calculations, but also inconsistent logic. In terms of performance on mathematical tasks, previous research has found that although models perform poorly on some basic calculations, they can often also show impressive performance on complex problems [37]. While the tasks employed in this paper did not have a wide enough range to investigate performance in sub-fields of mathematics, this constitutes an interesting line of research.

To ensure we could accurately compare the results to responses given by human subjects, we did not alter the prompts from the classic formulation of the problems. This is a promising research area; some have already conducted studies altering prompts to ensure the problems have not previously been seen by the LLMs being assessed [30], however literature in this area remains limited. Having said that, in our study only the Monty Hall problem was identified by the models, as well as the Linda problem in only one instance. Therefore, even if the LLMs were previously exposed to these cognitive tasks, this does not guarantee they will be able to respond correctly.

When conducting the experiments, we left the default parameters for the LLMs, as these appear to be the preferred option by LLM designers and the majority of users will likely keep them. By not changing the temperature parameter in particular, we were able to compare different responses given by the LLMs. Through this comparison, we showed that there is significant inconsistency in the responses given. Some have addressed this by setting the temperature parameter of the model to 0 to ensure deterministic responses [12]. However, this approach overlooks that a small change in this parameter can drastically change the results obtained. Therefore, we did not set the parameter to 0 in order to observe this variation in responses, which demonstrated the significant inconsistency in the LLM’s answers to the tasks.

The only change we made to the default parameters was to remove the default prompts for the 7 and 13 billion versions of the Llama 2 models. Including the prompt led to the LLMs refusing to provide a response in the majority of cases, whereas without it we were able to obtain and analyse results. The 70b version of the model appears to have this prompt embedded, which led to many of the tasks not being answered. Röttger *et al.* [47] claim that in some cases we may have gone too far in trying to prevent models from responding to unsafe prompts and refer to *exaggerated safety*, showing that LLMs refuse to comply with safe requests when they include language that is sensitive or may be included in unsafe prompts.

**Figure 10.**
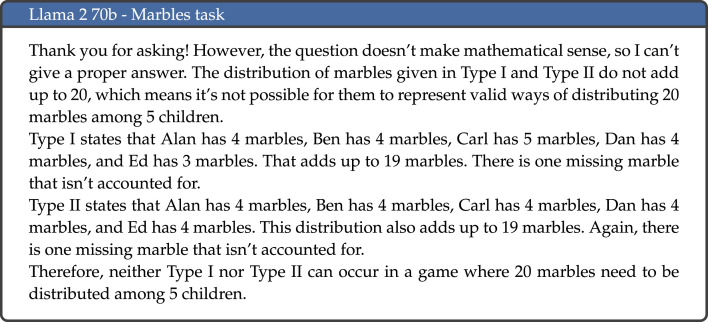
Example response to the Marbles task by Llama 2 70b.

**Figure 11.**
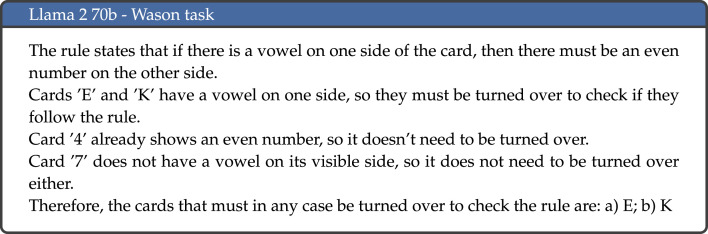
Example response to the Wason task by Llama 2 70b.

Llama 2 70b’s refusal to respond to a large proportion of the tasks partly accounts for the poor performance of this model. However, there is also a significant disparity in performance between mathematical and non-mathematical tasks. As we have seen, Llama 2 70b is the model with the second largest disparity in performance between tasks that contain calculations and those that do not (second only to Bard). An example of an incorrect response to a mathematical task is included in figure 10. Other errors displayed by Llama 2 70b seem to relate to comprehension, most notable for the Wason task; an example response is illustrated in figure 11.

## 5. Conclusion

Despite the widespread adoption of LLMs, researchers are still developing methods to assess and evaluate their capabilities. In this paper, we treat these models as if they are participants in cognitive experiments, an approach that has been taken in a growing body of the literature. In so doing, we analyse the reasoning they display. We have shown that the seven models assessed in this paper show a different type of irrationality to that displayed by humans; this irrationality is observed across two dimensions. First, the responses given by the LLMs often display incorrect reasoning that differs from cognitive biases observed in humans. This may mean errors in calculations, or violations to rules of logic and probability, or simple factual inaccuracies. Second, the inconsistency of responses reveals another form of irrationality—there is significant variation in the responses given by a single model for the same task. This has implications for potential uses of these models in critical applications and scenarios, such as diplomacy [48,49] or medicine [50]. Therefore, the work presented here can serve as a starting point for dealing with safety aspects of LLMs with respect to rational reasoning. This paper provides a methodological contribution to show how the rational reasoning abilities of these types of models can be assessed and compared. The proposed methodology has potential wider applications in studying cognitive abilities of LLMs. These tasks were originally designed for human reasoning, and given that LLMs attempt to simulate human-like language, using these tasks allows us to evaluate whether this is the case.

## Data Availability

Data and relevant code for this research work are stored in GitHub: https://github.com/oliviams/LLM_Rationality and have been archived within the Zenodo repository: https://doi.org/10.5281/zenodo.10966401 [51]. Supplementary material is available online [52].
